# The Association among Emotions and Food Choices in First-Year College Students Using mobile-Ecological Momentary Assessments

**DOI:** 10.1186/s12889-018-5447-0

**Published:** 2018-05-02

**Authors:** Jessica Ashurst, Irene van Woerden, Genevieve Dunton, Michael Todd, Punam Ohri-Vachaspati, Pamela Swan, Meg Bruening

**Affiliations:** 1 College of Health Solutions, Arizona State University, 550 N 3rd Street, Phoenix, AZ 85004 USA; 2 Institute for Health Promotion & Disease Prevention, University of Southern California, SSB 302E 2001 N. Soto Street Health Sciences Campus, Los Angeles, USA; 3 College of Nursing and Health Innovation, Arizona State University, 500 N 3rd Street, Phoenix, AZ 85004 USA

**Keywords:** Ecological momentary assessment, Emerging adults, Eating behaviors, Emotion

## Abstract

**Background:**

Studies have examined the associations between emotions and overeating but have only rarely considered associations between emotions and specific food choices. The purpose of this secondary data analysis was to use mobile ecological momentary assessments (mEMAs) to examine associations between emotions and food choices among first-year college students living in residence halls.

**Methods:**

Using an intensive repeated-measures design, mEMAs were used to assess concurrent emotions and food choices in a racially/ethnically diverse sample of first-year college students (*n* = 663). Emotions were categorized as negative (sad, stressed, tired), positive (happy, energized, relaxed), and apathetic (bored, meh). Assessments were completed multiple times per day on four quasi-randomly selected days (three random weekdays and one random weekend day) during a 7-day period using random prompt times. Generalized estimating equations (GEE) were used to examine between- and within-person associations of emotional status with a variety of healthy and unhealthy food choices (sweets, salty snacks/fried foods, fruits/vegetables, pizza/fast food, sandwiches/wraps, meats/proteins, pasta/rice, cereals), adjusting for gender, day of week, and time of day, accounting for within-person dependencies among repeated measurements of eating behavior.

**Results:**

At the between-person level, participants who reported positive emotions more frequently compared to others consumed meats/proteins more often (OR = 1.8; 99% CI = 1.2, 2.8). At the within-person level, on occasions when any negative emotion was reported (versus no negative emotion reported) participants were more likely to consume meats/proteins (OR = 1.5, 99% CI = 1.0, 2.1); on occasions when any positive emotion was reported as compared to occasions with no positive emotions, participants were more likely to consume sweets (OR = 1.7, 99% CI = 1.1, 2.6), but less likely to consume pizza/fast food (OR = 0.6, 99% CI = 0.4, 1.0).

**Conclusions:**

Negative and positive emotions were significantly associated with food choices. mEMA methodology provides a unique opportunity to examine these associations within and between people, providing insights for individual and population-level interventions. These findings can be used to guide future longitudinal studies and to develop and test interventions that encourage healthy food choices among first-year college students and ultimately reduce the risk of weight gain.

## Background

The transition from adolescence to young adulthood, or “emerging adulthood,” is said to be an especially vulnerable period for excess weight gain [1, 2]. During this critical time period, many emerging adults in college make poor food choices [2–6], which may affect long-term health outcomes [7], particularly among first-year college students.

The transition into college is also an emotional experience for emerging adults [8]. Stress is a commonly cited negative emotion among college students [8–11] and is frequently associated with emotional eating [8–12]. Emerging adults have identified negative emotions as contributors to their own overeating [3]. When feeling stressed, they may turn to food for psychological comfort [10, 12]. There is a significant body of literature to support that stress is linked to binge eating [13–15]. however, the causal relationship is still unknown [15], even in this more developed area of the field. At the same time, research suggests that for some, stress may not change eating habits [16], or may even cause decreases in eating [17]. Eating in response to stress is characterized by eating more food and/or consuming more calories [10], though there is only limited evidence regarding which types of foods are consumed from various food groups. Some research indicates that the foods people eat when experiencing stress are likely to be energy-dense, specifically high in sugar and fat [10, 18, 19]. For instance, a study examining female college students found that over half of participants reported an increase in appetite from stress, and those participants chose to consume significantly more types of sweet foods and mixed dishes which included high-fat items like casseroles, burgers, pizza, and fast food [10]. In these studies, stress was the only emotional aspect assessed, and healthy food choices were not examined.

Negative emotions that have been studied in relation to eating include sadness, anger/frustration, anxiety/fear, and boredom [20–22]. Some have argued that boredom should not be considered a negative emotion, but rather its own distinct emotional state, especially in relation to eating [23, 24]. Indeed, in open-ended questionnaire items, emerging adults in college have reported eating more in response to boredom than in response to negative emotions like anger and anxiety [24].

Certain gaps and inconsistencies remain in the study of emotion-related eating. For example, positive emotions are rarely mentioned in the eating behavior literature. Although, the very small group of studies that have examined positive emotions indicate that positive and negative emotions do differ in their associations with specific eating behaviors [8, 21, 22, 25]. In general, negative emotions are linked to adverse eating behaviors (e.g., overeating), while happiness is not associated with changes in eating behaviors [8]. Further, the motivation to eat is higher for negative emotions like anger and tension than for positive emotions like joy and relaxation [22]. Of the studies examining associations between emotions and eating, no studies have included a sample of first-year college students who happen to be at risk for weight gain and changes in their eating behaviors, and only a few studies have addressed associations between emotions and *types of food* consumed [10, 12, 25–27]. While not first-year students, a study of low-income adolescents found that consuming both sweet and salty snacks as well as sweetened beverages were associated with feeling lonely or bored [28]. The majority of these studies have used retrospective surveys for data capture [10, 12, 25, 26], though, Oliver et al. conducted a study experimentally manipulated stress (resulting in negative mood) and found higher intakes of sweet high-fat foods and a more energy-dense meal [27]. A meta-analysis examining effects of positive and negative moods on food choices and eating behaviors in laboratory settings, suggested that negative mood was associated with greater food intake, while positive mood was linked to higher caloric intake [29]. Retrospective self-reports and laboratory studies fail to capture the temporal associations between rapidly unfolding phenomena like the experience of emotion and food choices in ecologically valid settings. As such, these methods pose limits on understanding under what circumstances first-year college students make particular food choices.

Ecological momentary assessment (EMA) is a data collection technique in which a participant’s thoughts, feelings, and behaviors are repeatedly assessed in real time or near-real time in the contexts which they are occurring [30, 31]. Thus, EMA mitigates concerns regarding retrospection-related biases and lack of ecological validity. Historically, EMA studies of health behaviors have used palmtop computers [13, 32–34] and, more recently, mobile phones [31, 35, 36] allowing for self-reported assessment of health behaviors as they occur naturally in real life situations [35, 37, 38]. EMA via mobile phones (mEMA) appears to be a feasible and acceptable strategy for measuring physical activity behaviors [33, 35]. As yet, links between emotions and food choices have been assessed only in a limited fashion via mEMA methods. Given that first-year college students are vulnerable to weight gain and unhealthy lifestyle habits, our study aimed to use mEMA methods to determine associations among negative, positive, and apathetic emotions and food choices in first-year college students. Our specific hypotheses for this study were:

1. When first-year college students report negative emotions they will tend to consume more sweets, salty snacks/fried foods, and pizza/fast food.
2. When first-year college students report positive emotions they will tend to consume more fruits/vegetables.
3. When first-year college students report apathetic emotions they will tend to consume more sweets, salty snacks/fried foods, and pizza/fast food.

The results will provide an understanding of these relationships in the context of how emotions might be involved the healthy and unhealthy food choices that are occurring so that healthy food choices can be better promoted in this vulnerable population of emerging adults.

## Methods

### Study design

A multi-wave intensive longitudinal design employing mEMA methodology was used to capture associations among emotions and food choices in first-year college students living in residence halls on campus. This study was a secondary data analysis from the SPARC study [39], a larger, longitudinal study assessing the social impact of nutrition and physical activity in first-year college students.

### Participants and setting

The SPARC study drew on of an opportunistic sample of 1435 college students. Inclusion criteria were first-year students enrolled at the university during the Fall 2015 semester. Android or iPhone smartphones were required for students to complete mEMA questionnaires. Students who did not own an Android or iPhone but met all other criteria were provided with loaner phones to use for the study.

Recruitment took place during weekly floor meetings at six different residence halls at a major university in Arizona. All participants who chose to participate in the study provided written informed consent during recruitment. mEMA data collection was carried out in four waves during the school year; the data for this analysis are from the first wave only, collected in October 2015. Participants were incentivized for their continued participation at each mEMA data collection time period with $5 gift cards provided for every 10 completed mEMA reports. For each 7-day mEMA data collection period, an additional incentive of $5 was offered for completing at least 75% of that period’s assessments. More details on the study protocols can be found elsewhere [39]. The analytic sample for this study included those participants who reported eating before responding to at least one of the mEMA prompts (*n* = 663).

### Data collection

A custom mEMA application, “devilSPARC” was developed for this study [39]. Participants were asked to download the devilSPARC app onto their smartphone and then “opt in” to a text messaging service provider; participants were trained on site and received a handout on what to expect from the mEMA. Participants received prompts via the text messages to complete the surveys during four quasi-randomly selected days throughout a 7-day period (three randomly selected weekdays and one randomly selected weekend day), eight times per day, between 9 am and 10 pm (totaling 32 prompts across the mEMA data collection period). Each prompt contained a link in the text message which opened the devilSPARC app. Seven of each day’s eight assessments asked participants’ current behavior, and one asked about behavior over the previous three hours. Only the reports of current behavior were included in these analyses. Under a signal-contingent prompting schedule, prompts were sent to participants twice at randomly selected times during each of the following time windows: 9 am to 12 pm, 12 pm to 3 pm, 3 pm to 7 pm, and 7 pm to 10 pm. Participants had up to 30 min to respond to the prompt; when participants did not respond within the 30 min the assessment was closed and the prompt was recorded as missed. Each assessment took approximately one minute to complete.

Once the app was opened, a personalized welcome screen appeared with the participant’s name advising them to begin the assessment. The first question in the assessment examining current behavior was, “What were you doing right before you got this text? (please check all that apply).” The answer choices included eating, drinking, being physically active, or none of the above. Only assessments in which “eating” was endorsed were used for this analysis (*n* = 2209 observations).

The next question asked: “What are you eating? (please check all that apply).” Participants made selections from food group categories that included the following: cookies, sweetened baked goods, candy and frozen desserts; salty snacks and fried dishes; fruits and vegetables (including salads); pizza and fast food; sandwiches, wraps, breads, pitas, and tortillas; meat, poultry, fish, eggs, and meat alternatives; pasta, noodles, rice, and other grains; and hot and cold cereals. These options were developed based on formative work by Laska et al. with college students, which identified common food groups consumed: 1) cookies and sweetened baked goods, 2) candy and gummy fruit snacks, 3) salty snacks, 4) fruits and vegetables, 5) frozen desserts, 6) non-milk dairy products, 7) entrees, 8) cereals and grains, and 9) fried side dishes [40]. These food groups were adapted to after our pilot with first-year college students [41]. Participants could select more than one food choice. An information button was available for participants to use if they were unsure of which category to choose, and this provided them with a more detailed description of the food category (e.g., *Salty snacks including potato chips, veggie straws, cheese-flavored crackers, beef jerky, popcorn, pretzels, nachos, or string, sliced, shredded, or wedged cheese*.). Each food group category was analyzed separately for this analysis.

After the participants had selected their food choices, they were asked about their emotions with the question “Just before I started eating, I was feeling…” The response options were: happy, hungry/thirsty, tired, bored, meh, energized, relaxed, sad, stressed/nervous/anxious, sick, none of the above, or other. These options were the most common responses during our formative work and adapted after pilot testing with first-year college students [41]. Participants could select more than one emotion. If a participant’s current emotional state was not included in the list, he or she could choose “other” and manually write in a response. The “other” responses were examined and back-categorized as appropriate, as many of these fit into existing emotions; any remaining-open ended text responses were not analyzed due to small numbers. Responses endorsing non-emotional feelings (hungry/thirsty and sick) were not included in this analysis. The responses were then categorized into three groups (positive, negative, and apathetic based on the circumplex model of affect, where low arousal responses (e.g., “meh”) were classified as apathetic.

Dichotomous composite indicators of positive, negative, and apathetic emotions were created based on responses to individual emotion items. A measurement occasion when at least one positive emotion (happy, energized, or relaxed) was recorded was coded as 1 on the positive emotion indicator, otherwise, the occasion was coded as 0 on this indicator. The negative emotion and apathetic emotion indicators were created in the same way using their corresponding mEMA items (sad, stressed/nervous/anxious, or tired; and bored or “meh”, respectively). Next, person-level means for the composite emotion indicators were computed by taking number of occasions a person reported each type of emotion and dividing that by the number of measurement occasions from that person that were available for analysis. The person-level means were centered by the group mean proportion. These means reflect the proportion of occasions when a person reported a given type of emotion and capture the between-person portion of the variation in the emotion indicators. Then, each participant’s person-level mean on each emotion measure was subtracted from each occasion’s score (either 0 or 1) on that measure, yielding a person-mean centered version of the emotion type indictor (e.g., for a person reporting negative moods on 25% of measurement occasions the person-level mean would be 0.25. As the prompt scores were either 0 or 1, the person-mean centered negative emotion measure would take on values of either − 0.25 or 0.75). These person mean-centered variables capture the within-person portion of the variation in the emotion type measures.

This analysis examined measurement occasions when negative emotions, positive emotions, and/or apathetic emotions were reported. Self-reports of sex, race/ethnicity, and Pell grant status (a Federal grant for low-income students) were also collected at study intake. Race/ethnicity was categorized as either non-Hispanic White, non-Hispanic Black, Hispanic, or other.

### Data analysis

Associations between sociodemographic factors and compliance with the mEMA protocol were examined using general linear models (GLMs). Generalized estimating equations (GEEs) were then used to estimate the between- and within-person associations between emotions (positive, negative, and apathetic) and food choice categories. In preliminary analyses, sex, race/ethnicity, Pell grant status, residence hall, day of week, and time of day were examined as potential confounders and day of the week and time of the day were considered as potential effect modifiers. None of the model terms involving race/ethnicity, Pell grant status, or residence hall were significantly related to food choices. Accordingly, a more parsimonious model, adjusting only for sex, day of the week, and time of day, was used for the main analyses. We examined day of the week and time of the day as effect modifiers; none of the interactions were significant; as such, we adjusted the models for day of the week and time of day. All three dichotomous occasion-level indicators of emotion (any positive, any negative, and any apathetic) and all three person-level measures of emotion were considered simultaneously in the models. Each of the food choices were analyzed separately as presence versus absence (e.g., consumption of sweets versus not). Estimates of between-person associations characterize the relationship between the person-level frequency of reporting given emotion (i.e., the proportion of measurement occasions at which a person reported the emotion) and the person-level frequency of reporting the food choice of interest. That is, they address the question: Do people who experience a particular emotion relatively more often tend to make the food choice of interest more (or less) frequently than those who experience that emotion less frequently? Estimates of within-person associations characterize the odds that the food choice of interest would be made on an occasion when a given emotion is experienced.

Because the response variables (food choice measures) were dichotomous, all models specified a logit link and binomial-distributed errors. To adjust for within-person non-independence (clustering) of the repeated observations from each person, repeated measurements of eating behavior were treated as being nested (clustered) within persons. Of the three within-person correlation structures considered (independence, unstructured, exchangeable), an exchangeable correlation structure yielded the best model fit. Because a large number of comparisons were made, statistical significance was fixed at *p* < 0.01. The statistical software R version 3.3.2 was used for all analyses, and GEEs were estimated using the geepack package [42].

## Results

### Descriptive characteristics

A total of 663 first-year students (mean age 18.4) participated in the first wave of mEMA data collection during October 2015. Participants completed an average of 19.3 mEMAs; after excluding retrospective assessments, this average decreased to 16.9. After excluding assessments where the participants did not report eating and those where incomplete information was provided, there were an average of 3.3 assessments per participant available for analysis; the vast majority of these surveys (99.6%) were excluded due to no eating behavior reported. A total of 2209 mEMA surveys (from 663 participants) examining current behavior with eating are included in this analysis. As with the full sample, there were more females (70.6%) than males in the sample (Table 1). Nearly half (48.9%) of participants identified as non-Hispanic White, 8.1% as non-Hispanic Black, 28.7% as Hispanic, and 14.3% as ‘Other’. More than one-third of participants (35.1%) reported being a Pell grant recipient. The average time between a prompt being sent and the prompt being answered was less than eight minutes. The compliance rate to complete at least one mEMA survey was 73.9%. Compliance with the mEMA protocol did not differ by Pell Grant Status or race/ethnicity; however, did vary by sex (data not shown): males were less likely than females to complete mEMAs (*p* < 0.001). Significant time of day and day of week effects were also observed (p < 0.001). Prompts sent in the morning and on the weekend had lower response rates (data not shown). Meats/proteins (29.2%) and fruits/vegetables (29.0%) were the two most commonly reported foods (Table 1). The food choices with the lowest prevalence were cereals (6.1%), pizza/fast food (14.4%) and salty snacks/fried foods (15.5%). The most common emotion type was positive (45.5%), followed by negative (31.4%), and apathetic (17.9%).

**Table 1:** Descriptive Statistics for Person-level Background Variables and EMA Variables (*n*=663 participants, 2209 surveys)^a^

Person-level measures (*n* = 663)	*N* (%)	
Gender (female)	468	(70.6)
Race/Ethnicity		
Non-Hispanic White	324	(48.9)
Non-Hispanic Black	54	(8.1)
Hispanic	190	(28.7)
Other	95	(14.3)
Pell grant recipient	233	(35.1)
	M (SD)	
Age	18.4	(0.5)
EMA variables (*n*=2209)	*N* (%)	
Food choices		
Sweets	446	(20.2)
Salty snacks/fried foods	343	(15.5)
Fruits/vegetables	640	(29.0)
Pizza/fast food	318	(14.4)
Sandwiches/wraps	492	(22.3)
Meats/proteins	646	(29.2)
Pasta/rice	425	(19.2)
Cereals	134	(6.1)
Emotions		
Sad	42	(1.9)
Stressed	179	(8.1)
Tired	574	(26.0)
Happy	692	(31.3)
Energized	196	(8.9)
Relaxed	397	(18.0)
Bored	177	(8.0)
Meh	270	(12.2)
Emotion types		
Negative (sad, stressed, tired)	694	(31.4)
Positive (happy, energized, relaxed)	1005	(45.5)
Apathetic (bored, meh)	396	(17.9)

^a^Participants were given the option to choose one or more emotions and one or more food choices

### Instances of concurrent emotions

**Fig. 1:**
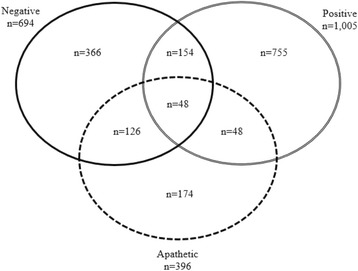
Counts of Emotion Combinations by Survey Among First-Year College Students (*n* = 663 participants, 2209 surveys). Single solid line indicates those surveys with negative emotion recorded. Double solid line indicates those surveys with positive emotion recorded. Single dotted lined indicates those surveys with apathetic emotion recorded. There were 538 surveys with no negative, positive, or apathetic emotion recorded

Given that participants could select more than one response on the mEMA surveys for emotions, there were several instances of concurrent emotions. These included survey responses with combinations of negative, positive, apathetic emotions. The number of survey instances for each emotion category as well as each emotion combination are presented in Fig. 1.

### Adjusted relationships between emotions and food choices

In GEE models mutually adjusting for positive, negative, and apathetic emotions, accounting for within-person clustering and for background covariates, the between-person results showed participants who reported positive emotions relatively more frequently had higher odds of consuming meats/proteins (OR = 1.8; 99% CI = 1.2, 2.8; Table 2) than those who reported positive emotions less frequently. There were no significant between-person associations between negative or apathetic emotions and eating behaviors.

**Table 2:** Generalized Estimating Equations Odds Ratios, (99% Confidence Intervals), and p-values for Between- and Within-Person Associations^a^ of Emotions with Food Choices

Emotion	Food choice	Between-person		Within-person	
		OR (99% CI)	*p*-value	OR (99% CI)	*p*-value
Negative^b^	Sweets	1.5 (0.9, 2.5)	0.037	1.3 (0.8, 1.9)	0.168
	Salty snacks/fried foods	1.2 (0.6, 2.2)	0.552	1.0 (0.6, 1.5)	0.844
	Fruits/vegetables	1.3 (0.8, 2.2)	0.168	1.1 (0.8, 1.6)	0.298
	Pizza/fast food	1.2 (0.6, 2.2)	0.470	0.8 (0.5, 1.2)	0.134
	Sandwiches/wraps	1.0 (0.6, 1.7)	0.905	1.1 (0.7, 1.6)	0.551
	Meats/proteins	1.3 (0.8, 2.1)	0.184	**1.5 (1.0, 2.1)**	**0.004**
	Pasta/rice	1.3 (0.8, 2.3)	0.164	1.4 (1.0, 2.1)	0.022
	Cereals	1.6 (0.6, 4.1)	0.230	1.1 (0.6, 2.4)	0.627
Positive^b^	Sweets	1.5 (0.9, 2.4)	0.042	**1.7 (1.1, 2.6)**	**0.002**
	Salty snacks/fried foods	0.8 (0.5, 1.4)	0.383	1.5 (1.0, 2.2)	0.015
	Fruits/vegetables	1.5 (1.0, 2.4)	0.012	1.4 (1.0, 1.9)	0.012
	Pizza/fast food	0.8 (0.5, 1.3)	0.238	**0.6 (0.4, 1.0)**	**0.007**
	Sandwiches/wraps	1.0 (0.7, 1.6)	0.817	0.8 (0.5, 1.2)	0.130
	Meats/proteins	**1.8 (1.2, 2.8)**	**<0.001**	1.2 (0.8, 1.6)	0.222
	Pasta/rice	1.6 (1.0, 2.5)	0.013	1.0 (0.7, 1.5)	0.903
	Cereals	2.0 (0.8, 4.8)	0.052	1.0 (0.5, 1.9)	0.890
Apathetic^b^	Sweets	1.2 (0.6, 2.3)	0.398	1.3 (0.8, 2.2)	0.151
	Salty snacks/fried foods	2.0 (1.0, 3.9)	0.013	1.4 (0.9, 2.2)	0.076
	Fruits/vegetables	0.7 (0.4, 1.4)	0.190	1.0 (0.7, 1.5)	0.766
	Pizza/fast food	2.0 (1.0, 4.1)	0.017	0.8 (0.5, 1.4)	0.387
	Sandwiches/wraps	1.0 (0.5, 1.8)	0.862	0.7 (0.5, 1.2)	0.083
	Meats/proteins	0.7 (0.4, 1.3)	0.163	0.9 (0.6, 1.3)	0.364
	Pasta/rice	0.7 (0.4, 1.5)	0.274	0.8 (0.5, 1.2)	0.182
	Cereals	1.0 (0.3, 3.1)	0.967	1.6 (0.7, 3.7)	0.136

^a^Associations were adjusted for sex, day of the week, time of day and within-person clustering of responses

^b^Negative emotions included sad, stressed, and tired; Positive emotions included happy, energized, and relaxed; Apathetic emotions included bored and meh

Boldface indicates statistical significance at *p*<0.01

Within-person results showed that on occasions with any negative emotion, participants were more likely to consume meats/proteins (OR = 1.5; 99% CI = 1.0, 2.1). On occasions with any positive emotion, participants were more likely to consume sweets (OR = 1.7; 99% CI = 1.1, 2.6) than on occasions with no positive emotion, but less likely to consume pizza/fast food (OR = 0.6; 99% CI = 0.4, 1.0). There were no significant within-person associations between apathetic emotions and eating behaviors.

## Discussion

This study examined how emotions were associated with food choices in first-year college students. Using mEMA methodology, this study yielded tests of within- and between-person associations between emotions and food choices, which is unique in the literature. Negative and positive emotions had unique relationships with food choices; apathetic emotions were not associated with any food choices. Characterizing the relationships among different types of emotions and food choices is helpful in understanding the motivations behind healthy and unhealthy food choices. These findings can be used to guide effective interventions for promoting healthy food choices among first-year college students while setting the stage for future longitudinal research to examine how food choices and emotions impact health outcomes over time among emerging adults. Additionally, these findings set the stage for future research to examine how under-and over-eating of certain food choices is related to positive, negative, and apathetic emotions.

First-year college students experiencing negative emotions may be less motivated to make healthy food choices and more likely to make poor food choices [10, 18, 19, 26, 27]. Eating behaviors are not solely determined by physiological needs. Theoretical frameworks of eating behaviors include the affect regulation model (e.g., coping with negative affect by consuming and sometimes overeating, unhealthy foods [43–45] or consuming healthy foods when experiencing higher, positive affect) [46] and the ego depletion model (e.g., in stressed situations, one is unable to exert self-control, thus consuming, and sometimes overeating, unhealthy foods) [47]. In the context of the current findings, more research is needed to understand the psychological contexts that predict eating choices. For example, the current study found that on occasions when participants reported negative emotions, they were more likely to report consuming meats/proteins. Food is often used as a distraction to draw one’s attention away from their negative emotions [8]. Given that meats/proteins tend to be high in fat and tend to be easily overconsumed [10], our finding provides a new ecological understanding of why first-year college students may be making unhealthy food choices during their first year on campus. These data can be used for future studies to examine how food choices and emotions are related to the development of overweight and obesity that occurs during the first year of college [5, 48–50].

Our study found participants who reported positive emotions relatively more often than their peers were more likely to consume meats/proteins. Participants were also more likely to report the consumption of sweets, and less likely to consume pizza/fast food, on occasions when they reported positive emotions. The association of lower consumption of pizza/fast food is consistent with one previous retrospective study which found that more “healthful” foods and less “junk” foods were consumed during positive emotions [25]. However, the increased consumption of sweets was also positively associated with positive emotions which is not consistent with the retrospective study findings [25]. In focus groups, college students have said that making healthy food choices can be difficult because of time constraints, reliance on pre-cooked meals and unhealthy foods served on campus, and not knowing how to prepare healthy foods for themselves [51, 52]. Given that emotions are rarely examined in the context of healthy eating, more research is needed to replicate the current findings. If indeed emotions are related to healthy food choices, helping students to be more mindful of their emotions may promote overall greater nutritional status.

Apathetic emotions were not related to first-year college students’ food choices. Previous studies have reported that boredom may be a frequent reason for snacking in college students [53, 54]. Snacks often consumed by college students consist of chips, crackers, and nuts [53], which are generally salty and/or fried. Given that snacking contributes significantly to weight problems in college students [54] and because apathetic emotions and foods choices have never been assessed among this population using mEMA methodology, it will be imperative that we continue to assess apathetic emotions and food choices to confirm these novel findings, and use this new information to create interventions that will educate first-year college students on how to avoid and/or make better use of their apathetic emotional experiences. For example, because this study found that positive emotions are positively associated with choosing sweet foods, future research may consider investigating whether strategies to temporarily regulate affect to more neutral states would sometimes be beneficial to overall eating behaviors.

This study is the first to examine within and between person associations of positive, negative, and apathetic emotions and food choices. Interestingly, there were few similarities in statistically significant findings across the within and between person findings. We observe a few more within-person findings than between person findings, suggesting that the effects are acute, short-term, and transitory in nature (play out on a micro-timescale), particularly when reporting positive emotions in relation to food choices. To date, most studies examining emotions and eating behaviors have focused on differences between individuals [8–23]. While these current findings need to be replicated, future research may consider the examination of why we see more variation at the intra-individual level. Additionally, these findings indicate why it is important to examine relationships at varying levels of analysis.

There are a number of strengths that should be taken into account when considering the findings of this study. This study is the first to examine the associations among negative, positive, and apathetic emotions and a relatively large variety of food choices. Using mEMA instead of traditional retrospective surveys allowed for multiple, repeated, real-time assessments within the participant’s natural environment. This reduced the risk of retrospective recall bias and ultimately provided ecologically-valid data for this study. The app was developed in both android and iPhone operating systems so that participants used their own mobile phones to complete the mEMAs. Additionally, the large sample size and racial/ethnic diversity of the sample was another major strength, enhancing the generalizability of the findings.

The observational study design used here allows for examination of how variables covary together over time, it does not allow for causal inferences. Additionally, the data came from a convenience sample of first-year college students at a single university; therefore, the findings may not generalize to other emerging adults who are not first-year college students or to students in other universities. Eating choices were examined as groups of food based on previous formative work and were designed to keep the assessments brief [39]. However, the specific foods and the quantity of food within each group that were consumed is unknown; as such, we are unable to examine under- and overeating behaviors as they are associated with food choices.

Additionally, it is possible that with certain response sets (i.e., individual response patterns), some participants may have been more diligent and willing to complete the entire food and emotion item set (thus reporting more food choices and emotions experienced), while other participants may have rushed through the assessments (thus reporting fewer food choices and emotions experienced). Given that positive emotions were associated with both healthy and unhealthy food choices, individual response patterns may account for the observed findings. The measures in this study were self-reported, which could result in recall bias; however, mEMA methodology may have minimized this bias.

## Conclusions

Very little is known about how emotions are associated with food choices, especially in first-year college students. The findings of this secondary data analysis show that experiencing positive emotions more frequently than others is associated with an increase in consumption of meats/proteins between individuals. When participants experienced negative emotions, they were also significantly more likely report the consumption of meats/proteins; however, these results are significant for the within-person analyses only. When participants experienced positive emotions, they were significantly more likely to be associated with the report of the consumption of sweets and less likely to be associated with the report of the consumption of pizza/fast food in within person. Apathetic emotions were not associated with any of the food choices. The findings from this mEMA study add to the small body of literature that exists regarding emotions and food choices, providing a better understanding of the food choices that first-year college students make. Future research should continue to assess more specific food choices as well as how positive, negative, and apathetic emotions affect quantity of foods consumed. mEMA methodology provides a unique opportunity to examine these associations within- and between-people, providing insights for individual and population-level interventions. These findings can be used to develop and test interventions that encourage healthy food choices among first-year college students and ultimately reduce the risk of weight gain.
